# Live 4D-OCT denoising with self-supervised deep learning

**DOI:** 10.1038/s41598-023-32695-1

**Published:** 2023-04-08

**Authors:** Jonas Nienhaus, Philipp Matten, Anja Britten, Julius Scherer, Eva Höck, Alexander Freytag, Wolfgang Drexler, Rainer A. Leitgeb, Thomas Schlegl, Tilman Schmoll

**Affiliations:** 1 Center for Medical Physics and Biomedical Engineering, Medical University of Vienna, Vienna, Austria; 2 Carl Zeiss AG, Jena, Germany; 3 Carl Zeiss Meditec, Inc., Dublin, USA

**Keywords:** Medical imaging, Biophotonics, Imaging and sensing, Image processing, Machine learning

## Abstract

By providing three-dimensional visualization of tissues and instruments at high resolution, live volumetric optical coherence tomography (4D-OCT) has the potential to revolutionize ophthalmic surgery. However, the necessary imaging speed is accompanied by increased noise levels. A high data rate and the requirement for minimal latency impose major limitations for real-time noise reduction. In this work, we propose a low complexity neural network for denoising, directly incorporated into the image reconstruction pipeline of a microscope-integrated 4D-OCT prototype with an A-scan rate of 1.2 MHz. For this purpose, we trained a blind-spot network on unpaired OCT images using a self-supervised learning approach. With an optimized U-Net, only a few milliseconds of additional latency were introduced. Simultaneously, these architectural adaptations improved the numerical denoising performance compared to the basic setup, outperforming non-local filtering algorithms. Layers and edges of anatomical structures in B-scans were better preserved than with Gaussian filtering despite comparable processing time. By comparing scenes with and without denoising employed, we show that neural networks can be used to improve visual appearance of volumetric renderings in real time. Enhancing the rendering quality is an important step for the clinical acceptance and translation of 4D-OCT as an intra-surgical guidance tool.

## Introduction

Optical coherence tomography^1^ (OCT) is an imaging modality widely used in ophthalmology. By interferometrically measuring the depth-resolved back-scattering of partially coherent light, OCT enables high-resolution cross-sectional imaging of tissue. The combination of repeated axial scans (A-scans) along the transverse dimensions allows for two-dimensional B-scans and even volumetric imaging^2^. Nowadays, swept-source OCT (SS-OCT)^3^ can achieve A-scan rates in the MHz range^4^. Utilizing such high imaging speed, it is possible to acquire and visualize volumetric data in real-time, which is often called 4D-OCT^5–7^. As previously done for two-dimensional OCT^8^, it has been demonstrated that even 4D-OCT can be integrated into a surgical microscope, with the potential to enable increased precision of micro-surgical maneuvers^5^. Hence, 4D-OCT is a promising technology especially for ophthalmology, where it provides a new form of intra-operative visualization and guidance. However, fast acquisition speed comes at a cost of a lowered signal-to-noise ratio (SNR). The relatively high noise levels make the volumetric rendering more difficult and can, for example, lead to clutter, obscuring important details to the surgeon or making the visualization of details close to the noise floor impossible. Another source of image quality degradation in OCT is laser speckle, which is independent of the lowered SNR due to high imaging speed. It is caused by self-interference of scattered light and appears as dark and bright spots^9^, covering a large range of intensities. Despite carrying some information, speckle is commonly treated as noise in structural OCT imaging^10^. For diagnostic OCT, averaging of multiple spatially slightly displaced frames is a very simple yet effective method to reduce the noise level and yield extremely high quality scans. Especially advanced techniques such as speckle modulation^11^ or angular compounding^12^ can achieve high quality images without loss of resolution. However, the speed requirements as well as the dynamic scene limit the applicability of averaging in an intra-surgical scenario. It is therefore of great interest to reduce the level of noise in 4D-OCT systems by other means while preserving minimal latency as well as sufficient field-of-view (FOV) and volume rate. These demands, while dealing with data rates in the order of hundreds of megabytes or even gigabytes per second, currently impose great limitations to any data processing method.

To reduce noise in OCT images via post-processing, several classical algorithms have been proposed, including non-local filters such as block-matching and 3D filtering (BM3D)^13^, probability-based non-local means (PNLM)^14^, or shearlet-based filtering^15^. Several researchers have already shown that deep learning is a very promising approach to achieve high-quality image denoising, improving not only quantitative, but also perceived image quality rated by expert graders^16^. A major part in these perceived improvements is contributed by the smoothing of the speckled images.

Most, but not all of these approaches require paired training data, in most cases clean target images. With such data, several authors have demonstrated algorithms for substantial image quality improvements^17,18^, in some cases using generative adversarial networks (GANs)^19,20^. While these approaches allow for very good noise reduction, acquiring the required data can be difficult. To tackle this challenge, several self- or weakly-supervised learning methods have been developed. Many of these approaches are based on the idea that useful signal is deterministic while noise is random. In Noise2Noise^21^, a network is trained to predict one noise realization from another one, using two noisy acquisitions of the same scene. Since the noise is not predictable, Noise2Noise can lead to denoising performances comparable even to supervised training, which has successfully been shown also for OCT^22,23^, even in combination with the generation of super-resolution images^24^. Blind-spot networks like Noise2Void^25^ and Noise2Self^26^ have made it possible to learn denoising even from single instances. These self-supervised approaches are based on obscuring individual pixels in the training inputs, and learning to reconstruct the pixel values from the local surroundings. However, in their basic form, these algorithms fail if the noise is structured, i.e. not pixel-wise independent. This limitation was overcome by structured Noise2Void^27^ (sN2V), in which the spatial neighborhood of the blind spots is altered to remove any residual information about the noise in the relevant pixel from the input. Recently, Höck et al. proposed several adjustments to Noise2Void, which they termed N2V2, to reduce high frequency grid artifacts, including omitting the residual connection and the top-level skip connection in the U-Net architecture^28^.

So far, to our knowledge, real-time application of denoising networks in OCT has been limited to two-dimensional images (B-scans)^23,29^. Huang et al.^23^ adapted Noise2Noise and achieved frame rates of up to 64 Hz for B-scans of $512 \times 512$ pixels (px) denoised directly after acquisition with a spectral domain OCT at an A-scan rate of 70 kHz. Rico-Jimenez et al.^29^ have demonstrated the possibility to train a denoising network based on the idea of self-fusion, facilitating neighboring B-scans from volumetric data. In their approach, they trained a U-Net^30^ to estimate the local average of a total of seven registered B-scans from just three adjacent B-scans as input for every output image. With such a network implemented on a 200 kHz SS-OCT system, output frame rates of 22 Hz for B-scans with $512 \times 512$ pixels were achieved.

In this work, we go one step further and apply two-dimensional denoising extended to the three-dimensional regime and a real-time scenario for a MHz microscope-integrated OCT system with a more than two orders of magnitude higher voxel rate. Based on sN2V^27^ and N2V2^28^, we developed a self-supervised training method for denoising of OCT data using single noisy images as input, avoiding potential motion artifacts. With an adapted masking scheme to increase realism of the training data and a U-Net-based architecture^30^ specifically tailored to minimize computational complexity, a trained network was directly incorporated into the reconstruction software of a 4D-OCT prototype which is executed on a graphics processing unit (GPU). The main contributions of this work are:

- Denoising of two-dimensional OCT B-scans using blind-spot neural networks, demonstrating simultaneous improvements in denoising metrics and inference speed when omitting connections in the U-Net architecture in a setting with structured noise.
- The incorporation of a trained 2D convolutional neural network directly into a 4D-OCT prototype in a way which preserves the volume rate and does not increase latency by more than a few milliseconds.
- The demonstration of qualitative effects of denoising on volumetric renderings of the anterior and posterior segments of the eye.

## Methods

Our work is based on a previously published^31^ 4D-OCT system. We extended it by incorporating a neural network for noise suppression. The system properties are summarized in the first subsection, followed by a detailed description of the implementation of said network into the prototype. Afterwards, the self-supervised learning method for denoising of OCT images is described. Finally, evaluation criteria and experimental setups are outlined.

### 4D-OCT prototype

The SS-OCT system, which forms the basis of this work, is capable of 4D data acquisition and real-time rendering^31^. The sample arm of the interferometer is coupled to an ophthalmic surgical microscope (ARTEVO 800, ZEISS, Jena, Germany) with an add-on module for intra-surgical use. In the following sections, the basic properties of the system are introduced with special focus on the image reconstruction pipeline.

#### System properties

As light source, the OCT incorporates a tunable microelectromechanical systems vertical-cavity surface-emitting laser (MEMS-VCSEL) prototype (Thorlabs Quantum Electronics, Jessup, MD, USA) with a central wavelength of 1060 nm. The source has an optical power of 4 mW on the sample for all modes and a sweep repetition rate that can be switched between 100 kHz, 600 kHz and 1.2 MHz, with optical bandwidths of 102 nm, 75 nm and 97 nm, respectively. While the system is currently not used on human subjects, its optical power of 4mW on the sample was selected in compliance with laser safety standards^33–35^, provided laser power and scanner motion were continuously monitored.

The prototype can be operated in different acquisition modes for several applications. With the 100 kHz laser mode, it is possible to capture B-scans with an imaging depth of 29 mm, covering the entire eye length. Different 4D modes exist depending on the demands, with a trade-off between volume rate and field of view (FOV). For imaging of the anterior segment of the eye, a larger FOV is required, while for intra-surgical retinal imaging, as smaller FOV can be sufficient, allowing for a smoother display^5^. In the 4D scenario, spiral scanning^6^ with a constant step size between adjacent A-scans along the pattern is used. Additionally, because the spectral interferogram is recorded as a function of time, it can be split^32^ to double the effective A-scan rate while halving the bandwidth. Data are captured by an AlazarTech ATS9373 (Alazar Technologies Inc., Pointe-Claire, Canada) data acquisition card (DAQ). Volumes are split into several two-dimensional buffers along the spiral scan pattern, which are then processed. Important properties of the acquisition modes are summarized in Table 1.

**Table 1:** Acquisition modes of the 4D-OCT prototype^31^.

Imaging mode	2D	4D (anterior)	4D (posterior)
Spatial dimensionality	2D	3D	3D
Central wavelength	1060 nm	1060 nm	1060 nm
Total bandwidth	102 nm	75 nm	75 nm
Effective bandwidth	102 nm	37.5 nm	37.5 nm
Laser sweep rate	100 kHz	600 kHz	600 kHz
Effective A-scan rate	100 kHz	1.2 MHz	1.2 MHz
Field of view	14.9 mm	9.8 mm	4.5 mm
Imaging depth	29 mm	5 mm	5 mm
Axial resolution	5.9 µm	16.0 µm	16.0 µm
Lateral resolution	15.6 µm	15.6 µm	15.6 µm
Frame rate	40 B-scans/s	3 volumes/s	10 volumes/s
Single buffer size	$6656 \times 1300$ px	$1408 \times 9942$ px	$1408 \times 2352$ px
Buffers per instance	1	20	25
Time between buffers	25 ms	16.7 ms	4 ms
Voxels per second	$346.1\cdot 10^6$	$839.9\cdot 10^6$	$827.9\cdot 10^6$

For the 4D modes, doubling of the effective A-scan rate is achieved by spectral splitting^32^, halving the effective bandwidth. Resolutions are given as full width at half maximum (FWHM) in tissue.

#### Image reconstruction and display

To achieve the required throughput, the tasks of rendering and display are performed separately on two identical NVIDIA Titan RTX (NVIDIA, Santa Clara, CA, USA) GPUs^31^. Once acquired by the DAQ, buffers of predefined sizes (see Table 1) are transferred to the first GPU, on which the image reconstruction is performed. Representing 2-manifolds sampled from the imaged object, either along a straight line (2D mode) or spiral (4D modes), these buffers are treated as two-dimensional images. As a first step, a static background is subtracted and Hann smoothing is applied. Dispersion is compensated using a third degree polynomial^36^. Then, the fast Fourier transform (FFT) is applied, and the magnitude of the complex output is computed. Finally, the data are scaled logarithmically and a fixed gray value (black level) is subtracted to crop the noise floor. After reconstruction, the two-dimensional buffers are converted to 8-bit integers and mapped into a three-dimensional Cartesian grid. The entire processing pipeline is implemented in CUDA (version 11.6).

As soon as a volume is completed, it is transferred via NVLink to a second, identical GPU for rendering and display^31^. For real-time visualization, the software CAMPVis^37,38^ is used. Volumes are rendered by a given intensity-opacity transfer function, which can be adjusted within the software by setting the lower limit of intensities. The rendering opacity increases almost linearly with intensity, while being set to zero for weak signals below the adjustable threshold, or noise floor. Examples for such transfer functions as well as the effect of different settings on the renderings are shown in Fig. 6.

### Incorporation of a light-weight neural network into OCT-reconstruction

**Figure 1:**
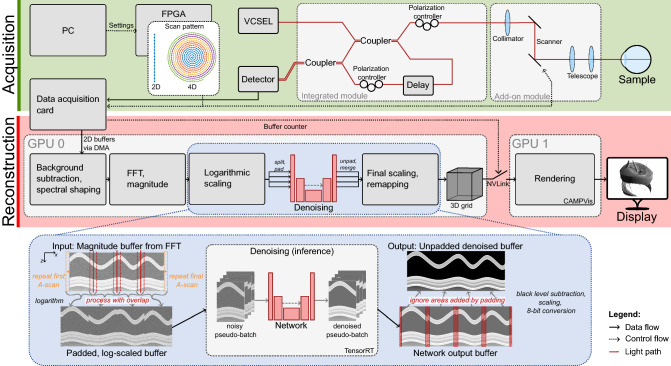
Overview of the 4D-OCT prototype including denoising. Denoising is incorporated into the processing pipeline of two-dimensional buffers, executed on a GPU. Size, number and spatial origin of buffers vary depending on the imaging mode, as indicated by different colors in the scan patterns. The buffer processing for denoising, including splitting into four sub-buffers and padding for pseudo-batching, is visualized in the bottom.

A neural network for denoising is directly incorporated into the data processing pipeline. Figure 1 gives an overview of the entire system, highlighting the position of the network. Network inference is performed on buffers after logarithmic scaling but before black level subtraction due to several considerations: Speckle, being multiplicative by nature^39^, becomes additive due to the logarithm, and can therefore be treated as additive noise, which better matches the assumptions of the denoising models. Furthermore, logarithmic scaling before denoising reduces the dynamic range requirements and increases interpretability of losses and evaluation metrics. Placement before black level subtraction and clipping makes the denoising less dependent on visualization settings. Furthermore, it can better preserve features close to the noise floor which would otherwise have already been cropped. The average of speckle noise after logarithmic scaling is assumed to be zero, in accordance to the commonly performed mean-preserving multi-frame averaging^40^. Denoising is employed on two-dimensional buffers instead on the final volume. While sacrificing potential advantageous effects from the full dimensionality, 2D convolutional networks are considerably less computationally expensive, and allow intermediate denoising of partial data instead of waiting for full volumes to be acquired, therefore reducing the introduced latency.

To increase throughput, buffers and the network are split into pseudo-batches by using 4 channels instead of a single one, as indicated in the bottom of Fig. 1. During export, the learned weights are copied for every channel, while setting all weights introducing channel interdependencies to zero. Despite the large overhead, we observed a considerable increase of the achievable throughput, possibly due to better utilization of the GPU’s tensor cores. Curiously, such a speed-up was not observed when using a network with a single channel and using the batch dimension instead. To avoid artifacts at the borders, the buffers are laterally padded by 20 A-scans at each side, accounting for the entire receptive field size of the network (as described below) of, in total, $41 \times 41$ pixels. At the lateral buffer edges, padding is performed by repeating the first and last A-scan respectively. For the intermediate lateral edges, which are introduced by the (pseudo-)batching, the true continuation is available and therefore used for padding, resulting in an overlap of the resulting images. After inference, the denoised pseudo-batch is recombined to a single buffer, by ignoring the overlapping region in the consecutive processing step, restoring the original buffer dimensions.

For our experiments, networks were trained in PyTorch^41^ 1.12.0, then exported first to ONNX 1.10.2 (opset version 16) and finally deployed using the TensorRT 8.4.0. C++ API. ONNX^42^ and TensorRT^43^ allow for different optimizations, of which fusion of convolutional layers, batch normalization and rectified linear units (ReLU) were enabled, as well as half precision (16 bit) floating point operations optimize the throughput. The modular TensorRT engines allow for large flexibility of the applied network.

### Self-supervised learning of a blind-spot denoising network

Commonly, clean training targets, generated by averaging of often 100 or more acquisitions, are used to train networks for denoising, either directly or by using GANs^17–20^. Instead, we focus on self-supervised training, relaxing the requirement for large amounts of paired training data. Assuming a clean pixel value $s$ is corrupted with random additive but not necessarily spatially uncorrelated noise $n$, a pixel value $v$ can be described as the sum of a signal and a noise component. The goal of denoising is to recover the uncorrupted signal $s$. The underlying idea of self-supervised denoising is that a network trained to estimate a pixel value $v$ can only predict the deterministic signal, but not the random, zero-mean noise contribution

1 $$\begin{aligned} E(v) = E(s+n) = \underbrace{E(s)}_{= s} + \underbrace{E(n)}_{=0} = s \end{aligned}$$

if it is ensured that no information about the actual noise realization is given to the estimator. In case of spatially uncorrelated noise, hiding this information can be achieved by simply masking the pixel of interest^25,26^. In (point-scanning) OCT however, the A-scan-wise acquisition as well as the nature of speckle cause spatial correlation of noise, such that hiding only a single pixel becomes insufficient.

#### Structured Noise2Void with continuous replacement

**Figure 2:**
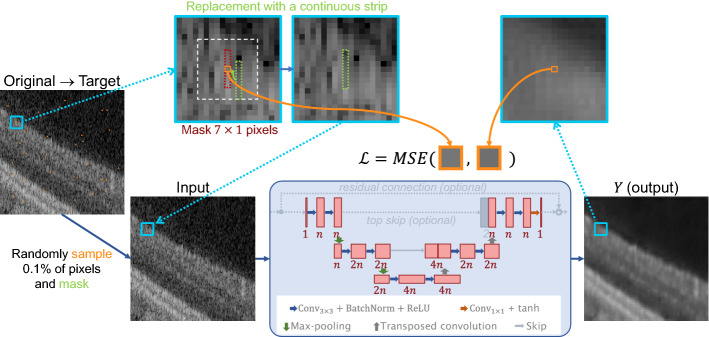
Proposed network architecture and training principle. Based on structured Noise2Void^27^, masking is adjusted such that neighbors are replaced together with center pixels. The principle is illustrated only for a single of multiple masked pixels. The network is a U-Net^30^ with no residual and top-level skip-connections. To control the complexity of the network, and therefore to set a trade-off between speed and complexity, channels $n$ of the individual convolution layers can be varied.

We adapt sN2V^27^ for self-supervised denoising. For training, a certain fraction of pixels is randomly sampled as center pixels. Based on the spatial noise correlation, certain neighboring pixels are masked as well to sufficiently remove noise information. In the published sN2V reference implementation^44^, the center pixels are replaced with random pixel values from the square local neighborhood, while the neighboring pixels are simply masked with uniformly distributed random values. This approach does not preserve any relation of the masked neighboring pixels to the local characteristics of the image. Instead, we propose to perform the masking by replacing the entire extended area with continuous strips from the neighborhood. For the experiments, a maximum offset of 5 pixels in each direction was used while allowing an offset of zero. The adapted sN2V masking scheme and network training principle is summarized in Fig. 2. In our experiments, we set the fraction of sampled center pixels to 0.1% and selected a mask size of $7\times 1$ to additionally mask the three adjacent pixels in either axial direction to the center pixels.

#### Network architecture and training

The network is a U-Net^30^, as shown in Fig. 2, tweaked specifically to accommodate the real-time requirements of our use case. For this reason, we limited the depth to two down- and upsampling steps, and use only operations which are supported by TensorRT^43^. Downsampling is performed via max pooling^45^ while transposed convolutions are used for upsampling. Both operations use kernels with $2\times 2$ pixels and strides of 2 to half or double the size of the feature maps. The number of filters $n$ in the uppermost level is treated as a tunable parameter to control the network complexity. For every deeper level, i.e. set of activation maps of the same image dimensionality, this number is doubled, such that there are $4n$ channels in the lowest layer. We adapted the omission of a residual connection and the top-level skip-connection proposed as N2V2^28^ to structured Noise2Void, to reduce high-frequency artifacts and—most importantly—the computational complexity. However, we did not adapt BlurPooling^46^ to keep the computational complexity low. The resulting network configuration is denoted as $\text {U-Net}^{\lnot r, \lnot t}$. To thoroughly evaluate the effect of the omission of the residual connection and the top level skip connection, we also trained network configurations with both a residual and a top-level skip connection ($\text {U-Net}^{r,t}$) in place, as well as only omitting the latter ($\text {U-Net}^{\lnot r,t}$).

Following the Noise2Void^25^ training regime, we defined one epoch as a fixed number of steps, while randomly extracting patches from the training images. One epoch consisted of 600 update iterations with a batch size of 128 patches with a size of $200\times 200$ pixels. The networks were trained for 300 epochs with Adam^47^ and an initial learning rate of $2\cdot 10^{-5}$. The mean squared error (MSE), calculated only for the sampled center pixels, was used as the loss with the original, unchanged value as target. Based on the loss on the validation data, the learning rate was halved whenever a plateau of 10 epochs was reached, and the network parameters with the lowest validation loss during training were finally exported. For further data augmentation, random horizontal flipping was used in 50% of the cases, essentially reversing the lateral scanning direction while preserving the A-scan-wise characteristics. Furthermore, we randomly varied the range of possible pixel values to emulate different levels of black level subtraction and dynamic range scaling. New upper and lower bounds were sampled from a normal distribution with zero mean and a standard deviation of $\sigma = 0.25$, but clipped to $\left[ -1,+1\right]$. Consequently, either bound remained unmodified with a probability of 50%, leaving the whole image unchanged in 25% of the cases.

#### Training and validation data

The training set consisted of multiple two-dimensional scans, most of which were extracted from volumetric acquisitions. From the 4D-OCT prototype, we used several different acquisitions of ex-vivo porcine and bovine eyes. The training set contained 2463 B-scans from the anterior segment of two pork eyes and 714 retinal B-scans from a cow’s eye. 511 anterior B-scans of another bovine eye and 996 of one porcine eye were reserved for validation. The animal eyes were obtained from local butcher shops.

Additionally, scans of healthy human retinas from both eyes of two subjects were included, while scans showing pathologic changes were reserved for numerical evaluation as described below. The study was approved by the Cleveland Clinic Institutional Review Board and conducted in accordance with the principles of the Declaration of Helsinki. Informed consent was obtained from all subjects. These data were acquired with a commercial PLEX Elite 9000 (ZEISS, Dublin, CA, USA), which is a SS-OCT system operated at a center wavelength of 1060 nm. It is therefore sufficiently comparable to the 4D-OCT prototype in terms of image characteristics, especially in terms of spatial noise autocorrelation relevant for the validity of the training method. All 2048 B-scans from one subject were used for training, while the 2000 B-scans from the second subject were reserved for validation.

Data from acquisitions and devices differed in subtracted noise floor and overall SNR. Examples from the training and validation datasets, along with individual noise autocorrelations, are given in the Supplementary material.

#### Numerical evaluation of two-dimensional denoising

For evaluation, the denoised images from a separate evaluation dataset were compared to ground truth images. Since the denoising method strongly relies on the spatial noise autocorrelation, it was crucial that the evaluation dataset had compatible characteristics. We used 18 ophthalmic diagnostic datasets from 11 different subjects, suffering from different diseases—in contrast to the B-scans from healthy subjects included in the training set. They were acquired with a PLEX Elite 9000 and each contained 100 B-scans which were registered and averaged for a ground truth. As input, a single noisy frame was used, which was also used as reference during registration and therefore unwarped, preserving realistic image properties.

Numerically, we evaluated the denoising performance based on structural similarity (SSIM)^48^ and peak signal-to-noise ratio (PSNR)

2 $$\begin{aligned} \textrm{PSNR}(Y,A) = 10\cdot \log _{10} \left( \frac{R^2}{\textrm{MSE}(Y,A)}\right) [dB] \end{aligned}$$

where $Y$ is the denoised image and $A$ the ground truth, $\textrm{MSE}$ is the mean squared error and $R=v_{max}-v_{min}$ the maximum intensity range, i.e. 255 for 8-bit integers (with $v_{min}=0$ and $v_{max}=255$). Additionally, for each noisy test image we defined $N_{ROIs}=14$ fore- and a single background region of interest (ROI) to evaluate the contrast-to-noise ratio (CNR)

3 $$\begin{aligned} \textrm{CNR}(Y) = \frac{1}{N_{ROIs}} \sum _{i=1}^{N_{ROIs}} \frac{\mu _{f_i} - \mu _b}{\sqrt{\sigma _{f_i}^2 + \sigma _{b}^2}} \end{aligned}$$

where $\mu _b$ is the mean and $\sigma _b^2$ the variance within the background ROI, while $\mu _{f_i}$ and $\sigma _{f_i}^2$ are mean and variance of the $i$-th foreground ROI.

For the final scores, PSNR, SSIM and CNR of all test cases were averaged, weighing all instances equally. Additionally, we report the double sample standard deviation of the individual scores. PSNR and SSIM were evaluated on crops of the images to remove potential registration artifacts at the edges and limit the background area. All 18 evaluation cases along with the cropped regions and ROIs are shown in Supplementary material.

Since real-time applicability is of particular interest, we also report average timings measured using NVIDIA Nsight Systems for profiling, as well as benchmark timings determined for inference without additional load during creation of the TensorRT-engines. Thresholds for stable real-time applicability were determined based on the empirical observation that no major parallelization of reconstruction steps on the single GPU could be achieved. Therefore, these thresholds were determined as the difference of the total time per buffer, as given in Table 1, and the average duration of all other reconstruction steps for processing a single buffer.

#### Baseline methods

The dataset does not contain any paired images, meaning that popular baseline methods such as Noise2Noise^21^ and supervised learning-based methods could not be applied. For this reason, we compared PSNR, SSIM and CNR to the non learning-based non-local filtering methods BM3D^13^ and PNLM^14^. For BM3D, the parameter $\sigma$ was empirically set to 80. Since our goal was real-time application, we selected a simple and fast Gaussian filter as additional reference, for which we empirically selected a standard deviation of 5 pixels which yields a good trade-off for the different denoising scores.

For timing of the reference Gauss filter in a comparable scenario, it was implemented in PyTorch as a single 2D convolution with a kernel size truncated to $21\times 21$ pixels, covering two standard deviations in each direction. The filter kernel was exported to TensorRT via ONNX following the exact same workflow as used for trained networks. Pseudo-batching was not applied here, since we did not observe any increase in speed for the Gauss filter. Like for the networks, we let TensorRT automatically determine^43^ the fastest CUDA-kernel, selected from options which included performing the discrete convolutions in time- or Fourier-domain and using single and half precision operations. The resulting method was a cuDNN-optimized^49^ spatial 2D convolution kernel without using half precision. To execute BM3D^13^ and determine its processing time as fairly as possible, we applied the publicly available CUDA-implementation BM3D-GPU^50^. This code was executed 100 times on images with sizes corresponding to the respective buffer sizes of the different imaging modes. For PNLM^14^, we used the official MATLAB-implementation, executed on an Intel i7 1165G7 CPU. Because of the dissimilarity in implementation, we report only very rough estimates of the PNLM processing times based on the run time of this implementation.

### Experimental evaluation of real-time denoising

For qualitative evaluation of the volumetric denoising, we imaged ex-vivo porcine eyes with and without denoising applied. The rendering transfer function was set to achieve a qualitatively good trade-off between preserved signal and removed noise for both settings. To improve comparability of the scenarios, a surgical instrument (Sinskey hook) was attached to a Meca500 robotic arm (Mecademic Robotics, Montreal, Canada) with a repeatability of 5 µm, which executed identical motion patterns in all compared scenarios. This motion pattern consisted of small push in the tool’s direction, followed by one or two eight-shaped patterns on a fixed axial plane or with small axial displacement before returning to the initial position. Additionally, we performed mock maneuvers manually. For retinal imaging, the porcine eyes were opened due to image quality reasons, keeping the vitreous mostly intact.

For documentation, we acquired screen captures of the live visualizations. These videos, as well as short descriptions and a depiction of the experimental setup, are available in the Supplementary material.

## Results

**Figure 3:**
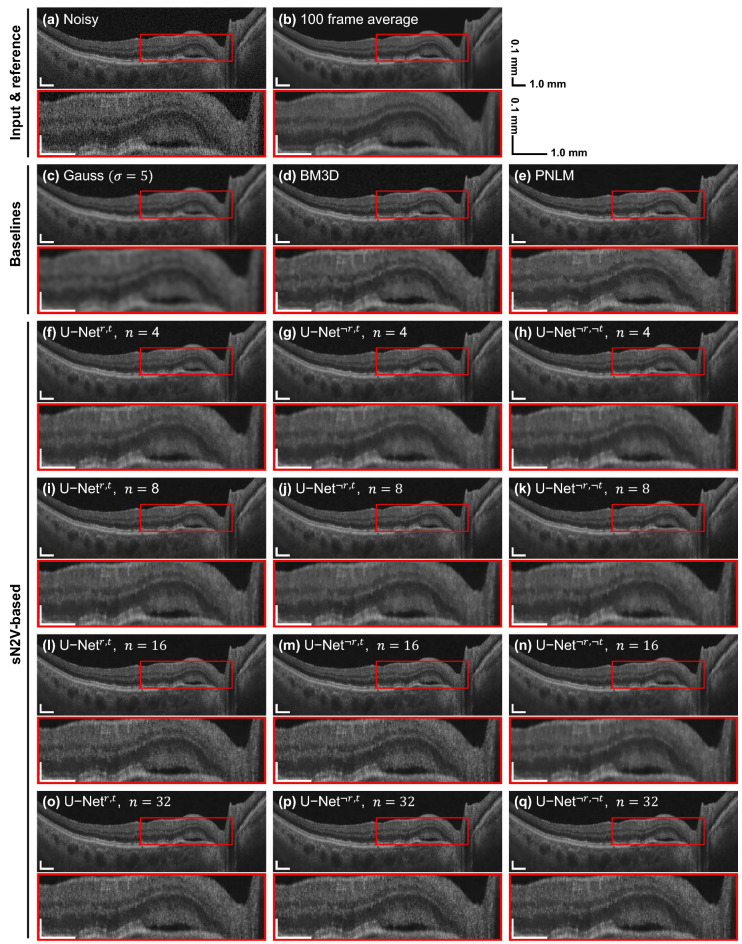
Example for 2D denoising of a B-scan from the evaluation dataset based on different denoising methods or network architectures. All networks were trained with an adapted sN2V^27^ strategy. More examples can be found in the Supplementary material.

As part of this work, we evaluated denoising performance and speed first on 2D data. Based on our findings, we then tested its application in live volumetric renderings. Findings and observations are summarized in the following sections.

### Denoising performance in 2D

**Figure 4:**
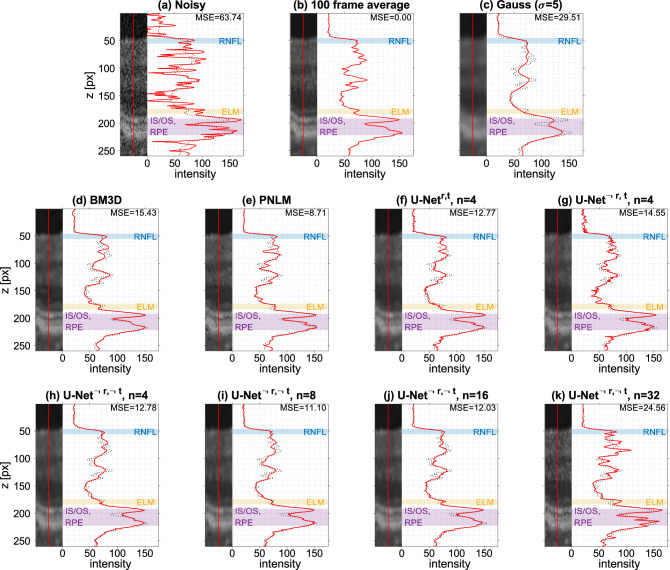
Intensity profiles along cropped A-scans at the center of the fovea for different denoising methods with multi-frame averaging as reference (dotted line) and highlighting of important retinal layers.

Figure 3 shows a comparison of denoised B-scans for different network configurations in comparison to multi-frame averaging and baseline filtering algorithms. Network configurations differed in complexity, controlled by the number of initial filters $n$, as well as presence ($r$) or absence ($\lnot r$) of residual connection and top-level skip connection ($t$ or $\lnot t$, respectively). The number of filters in the second ($2n$) and in the third level ($4n$) of the network were adjusted proportionally with $n$.

Qualitatively, deep-learning-based denoising reduced noise both in background and tissue areas. An increasing complexity and the usage of a residual or top-level skip connection are accompanied by a higher level of noise remnants in tissue regions, while background is not visibly affected. Compared to the Gauss-filtered B-scan, the images denoised by networks appear considerably less blurry, better preserving sharp surfaces and finer details. BM3D^13^ and PNLM^14^ could preserve edges better than the Gauss filter, but PNLM failed to completely remove speckle in the tissue. Differences in layer preservation are evident in axial intensity profiles across different retinal layers as shown in Fig. 4, plotted for the fastest versions of $\text {U-Net}^{\lnot r,t}$ and $\text {U-Net}^{ r,t}$ and all variants of the best-performing $\text {U-Net}^{\lnot r, \lnot t}$. In these profiles, sharper edges and higher inter-layer intensity differences are present with network-based denoising as well as BM3D and PNLM. Fine layers such as the retinal nerve fiber layer (RNFL) and the internal limiting membrane (ILM) as well as the contrast between different structures at the photoreceptor inner and outer segment junction (IS/OS) and the retinal pigment epithelium (RPE) are better preserved compared to Gaussian filtering. Accordingly, the MSE with respect to frame averaging along these axial lines is below 50% of that achieved with the Gauss filter, with an exception for the highest network complexity ($n=32$). When comparing the cropped regions, network configurations with a top-level skip connection ($\text {U-Net}^{\lnot r,t}$ and $\text {U-Net}^{ r,t}$) led to high-frequency axial stripes within tissue. These stripe artifacts are visible throughout different regions of the retina, especially in the bright IS/OS and RPE region. Such patterns are absent in the denoised B-scans when omitting both connections ($\text {U-Net}^{\lnot r,\lnot t}$, bottom row in Fig. 4), although similar patterns reappear for $n=32$.

**Table 2:** Denoising performance and timings for different methods.

Method/model			PSNR [dB]	SSIM	CNR	Per-buffer denoising time [ms]		
			$\mu$ $\pm ~ 2\sigma$	$\mu$ $\pm ~ 2\sigma$	$\mu$ $\pm ~ 2\sigma$	2D	4D (ant.)	4D (post.)
Original (unchanged)			$23.43$ $\pm ~ 1.04$	$0.237$ $\pm ~ 0.076$	$2.78$ $\pm ~ 1.45$	–		
100 acquisitions (averaging)			–	–	$8.31$ $\pm ~ 3.65$	–		
Gaussian filter (σ = 5 px) BM3D-GPU^50^ PNLM^14^			**35.44** ± 2.36	**0.930** ± 0.027	10.3 ± 6.46	6.4 (5.9)	11.0 (9.6)	2.3 (2.3)
			34.66 ± 2.50	0.913 ± 0.025	7.47 ± 4.48	638.5	973.1	283.1
			32.68 ± 4.21	0.858 ± 0.107	6.06 ± 3.00	> 1000*	> 1000*	> 1000*
sN2V-based	$\text {U-Net}^{r,t}$	$n=4$	$34.06$ $\pm ~ 2.04$	$0.862$ $\pm ~ 0.036$	$7.10$ $\pm ~ 3.32$	6.0 (6.2)	8.5 (9.8)	2.2 (2.3)
		$n=8$	$34.39$ $\pm ~ 2.30$	$0.881$ $\pm ~ 0.029$	$7.18$ $\pm ~ 3.66$	11.6 (12.9)	18.8 (19.0)	4.7 (4.4)
		$n=16$	$33.20$ $\pm ~ 2.81$	$0.856$ $\pm ~ 0.063$	$6.44$ $\pm ~ 4.42$	32.4 (34.0)	44.7 (50.8)	11.0 (11.3)
		$n=32$	$33.08$ $\pm ~ 3.01$	$0.852$ $\pm ~ 0.067$	$6.31$ $\pm ~ 4.43$	– (111.4)	– (164.1)	– (37.4)
	$\text {U-Net}^{\lnot r,t}$	$n=4$	$34.94$ $\pm ~ 2.38$	$0.904$ $\pm ~ 0.027$	$7.86$ $\pm ~ 3.86$	6.1 (7.3)	8.9 (10.6)	2.2 (2.6)
		$n=8$	$34.77$ $\pm ~ 2.47$	$0.902$ $\pm ~ 0.029$	$7.71$ $\pm ~ 3.90$	11.7 (14.4)	18.2 (21.2)	4.7 (4.9)
		$n=16$	$33.20$ $\pm ~ 3.13$	$0.862$ $\pm ~ 0.071$	$6.56$ $\pm ~ 4.97$	31.6 (37.3)	41.8 (54.7)	11.1 (12.6)
		$n=32$	$32.80$ $\pm ~ 3.55$	$0.855$ $\pm ~ 0.078$	$6.17$ $\pm ~ 4.85$	– (122.5)	– (171.8)	– (42.2)
	$\text {U-Net}^{\lnot r,\lnot t}$	$n=4$	$\underline{35.31}$ $\pm ~ 2.49$	$\underline{0.919}$ $\pm ~ 0.024$	$8.50$ $\pm ~ 4.31$	**5.6** (5.4)	**6.7** (6.9)	**1.7** (1.9)
		$n=8$	$35.23$ $\pm ~ 2.56$	$\underline{0.919}$ $\pm ~ 0.026$	$8.06$ $\pm ~ 3.93$	9.4 (11.2)	15.8 (14.6)	4.0 (3.9)
		$n=16$	$35.24$ $\pm ~ 2.71$	$0.916$ $\pm ~ 0.026$	$7.97$ $\pm ~ 3.81$	26.1 (29.5)	38.9 (44.1)	9.6 (10.5)
		$n=32$	$33.74$ $\pm ~ 2.95$	$0.884$ $\pm ~ 0.056$	$6.73$ $\pm ~ 4.72$	– (105.3)	– (148.8)	– (35.9)

**Figure 5:**
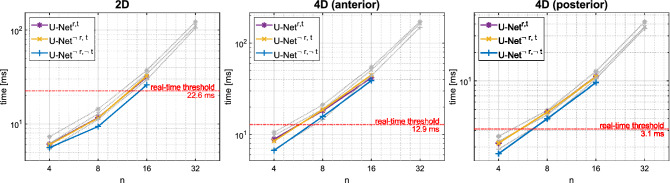
System and benchmark per-buffer denoising times for the three different imaging modes. All colored times, including the thresholds, were determined by profiling of the OCT software. Gray data points represent benchmark timings. For stable operation, processing times must be considerably lower than the real-time threshold, which is the available idle time of the GPU during reconstruction. Especially the omission of the top-skip of the U-Net increased the inference speed.

The comparison shows reference scores, as well as different network configurations for our adapted structured Noise2Void (sN2V). Where possible, timings were determined by profiling. Overall best PSNR and SSIM and execution times are marked in bold, best scores when only comparing different network setups are underlined. Note that different imaging modes have different timing requirements (see Fig. 5 and Table 1), and not all listed timings allow stable execution over an extended time period. Timings in parentheses were determined using the TensorRT-integrated benchmark on the same GPU (NVIDIA Titan RTX). Timings for PNLM^14^ ($^*$) could not be measured in a fair scenario, but exceeded seconds to even minutes when executed in MATLAB on a CPU.

Numerical scores, calculated on 18 pathological retinal B-scans, are shown in Table 2, along with corresponding processing times for individual buffers of the three different acquisition modes of our 4D-OCT prototype. The qualitative observations regarding image quality are largely confirmed quantitatively. In terms of PSNR and SSIM, the best performing networks clearly outperformed both BM3D^13^ and PNLM^14^. The highest scores were achieved with Gaussian filtering. However, the high CNR, far exceeding the value for averaged B-scans, is an indication of excessive blurring, as observed qualitatively.

When comparing network settings, lower complexities generally yielded superior performance in terms of PSNR and SSIM, while the CNR reached levels similar to multi-frame averaging. For higher network complexities, PSNR, SSIM and CNR dropped, underlining the observation of more noisy tissue appearance. This drop occurred later ($n=32$, compared to $n=16$) when dropping both the residual and the top-level skip connection. Generally, omitting the residual and top-level skip connection in the U-Net ($\text {U-Net}^{\lnot r,\lnot t}$) is associated with higher scores at every given complexity, indicating that these architectural changes can be beneficial not only in settings with spatially uncorrelated noise, as shown in N2V2^28^, but with structured noise as well.

### Real-time performance

For studying real-time applicability of the proposed denoising networks, achievable inference speed was of particular interest. Measured per-buffer denoising times are reported in Table 2 and visualized in Fig. 5 alongside the measured thresholds for stable real-time application in our 4D-OCT prototype. Sufficient speed was achieved only with a network size of $n=4$ initial kernels for both 4D modes, while $n=8$ initial filters were feasible for the 2D mode with its lower data throughput. Larger networks with slower inference led to increasing latency and unstable behavior. Omission of both the residual and top-level skip connection ($\text {U-Net}^{\lnot r,\lnot t}$) decreased the inference time by, on average, more than 15% compared to including both ($\text {U-Net}^{r,t}$). Leaving out only the residual connection ($\text {U-Net}^{\lnot r,t}$) only minimally influenced the measured timings. Notably, with similar deployment, Gaussian filtering was slower than the quickest network. Both BM3D^13^, even though executed on the GPU^50^, and PNLM^14^ did not nearly achieve the required throughput.

Due to the highest speed in combination with the best performance in terms of PSNR and SSIM, $\text {U-Net}^{\lnot r,\lnot t}$ is most appropriate for real-time use. Given the number of 20 or 25 buffers per volume, the total processing time of a single volume was 134 ms for the anterior and 42.5 ms for the posterior 4D mode. However, when buffers are denoised individually and a volume is rendered as soon as the final buffer is completely processed, the introduced latency is equal to the additional processing time of a single buffer only, which was 6.7 ms or 1.7 ms, respectively.

Since the implementation facilitated half precision (16 bit) floating point operations to increase speed, we analyzed its numerical effect on the output images. We observed median absolute errors of well below 0.1 gray values in 8-bit representation compared to single precision. Supplementary Video 1 shows a side-by-side comparison of live B-scans without and with denoising.

### Effect of denoising on volumetric real-time visualization

**Figure 6:**
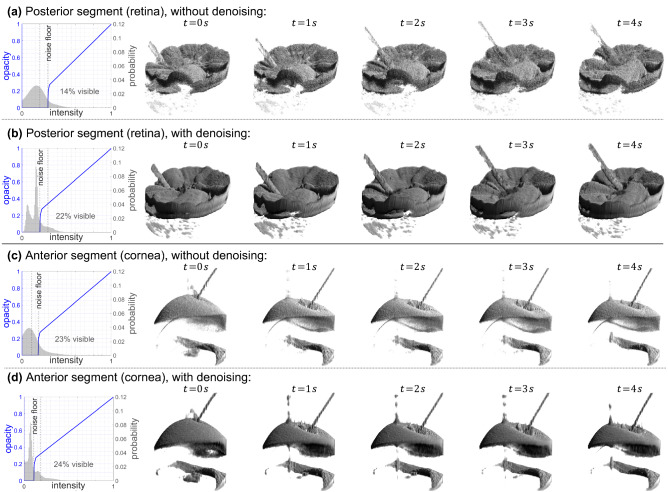
Frames showing volumetric renderings without and with denoising together with the respective rendering intensity-opacity transfer functions (blue curves) and gray value histograms. Each row shows a separate acquisition with different settings. The effect during real, dynamic acquisitions is best viewed in Supplementary Videos 2–4, which contain more examples as well as renderings using identical transfer functions.

The effect of the denoising applied to two-dimensional buffers during spiral-scanning volumetric acquisition is illustrated in Fig. 6 for the retina and the anterior segment of ex-vivo porcine eyes. The four volume series were captured separately with individual reconstruction and rendering settings, and manually synchronized with respect to robotic instrument movements. For every acquisition, the respective intensity-opacity transfer function, together with estimates for the gray value distributions of the volumetric data, is plotted on the left. Videos of these and more scenes with and without denoising and with different rendering transfer functions are provided as Supplementary Videos 2–4. In addition to the optimized renderings for each setting—with or without denoising—the videos also show recordings with the transfer function optimized for the other setting.

Even with an optimized transfer function, the rendering without denoising suffered from coarse surfaces caused by individual pixels falling beneath the noise floor, in particular due to speckle. With denoising, this effect was reduced due to a higher contrast between fore- and background and removal of speckle. This is especially visible for retinal imaging (Fig. 6a,b), where SNR was further reduced due to losses of light intensity caused by absorption and scattering in the vitreous. For the visualization of the retina, the visibility not only of surfaces, but also of the surgical instrument was greatly increased since more signal was preserved. The lower noise floor which can be used together with denoising indicates a higher potential dynamic range, corresponding to a higher range of rendered gray values as shown in the histograms in Fig. 6.

In all cases, a large fraction of the signal was discarded as background during rendering. Generally, this could be expected, since large portions of the data represented background which is not of interest for visualization. However, due to noise and especially (dark) speckles, a fixed gray value threshold led to removal of additional pixels and, ultimately, caused grainy surfaces. Denoising consistently allowed for a lower noise threshold, leading to more pixels being visible, which indicates a better signal preservation. Especially for the posterior segment, denoising considerably increased the fraction of rendered voxels from approximately 14% to 22%. For the anterior segment, approximately 24% were rendered compared to 23% without denoising. Notably, the gray value histograms after denoising contain narrow peaks. Both for the anterior and posterior segment, the most prominent of these peaks fall below the rendering noise floor and correspond to noise in the upper or lower regions of the B-scans, respectively.

Comparison of Fig. 6c,d shows that denoising in combination with the adjusted transfer function increases the impact of hyper-reflection artifacts, which occur due to detector saturation in A-scans with approximately horizontal corneal surfaces. When investigating these artifacts in dynamic scenes and at identical transfer functions as shown in Supplementary Videos 3 and 4, it is revealed that this impact is largely caused by the lower noise floor. Other than noise, artifacts are not explicitly removed and therefore preserved in the output signal.

Potential improvements of within-tissue details, as visible in 2D scans, could not be observed due to the nature of the three-dimensional rendering, which obscures these deeper layers. These were only visible at the edges of the rendered volume, where the removal of bright speckles led to an apparent reduction of tissue layer contrast.

## Discussion

For the first time, we have shown that high resolution B-scans from an OCT system with A-scan rates in the MHz regime, and based on that even 4D-OCT data, can be denoised in real-time using a neural network, leading to considerable improvements in the perceived quality of the live visualization. Without requiring any paired training data, noise levels are reduced in tissue as well as background regions, showing effectiveness of the proposed method for the different types of noise, including speckle. As a consequence, volumetric visualizations profit from much clearer surfaces. Because of the on-the-fly denoising of individual buffers, the introduced latency is considerably shorter than the acquisition time of each volume. Such low latency is crucial for intra-surgical use. In combination with strongly improved visibility of details and image quality in general, our work may greatly increase usability of real-time volumetric OCT and its acceptance by surgeons.

Inter-layer contrast appears stronger in images denoised by a network compared to filtering, but still weaker than in averaged frames. Bright spots in B-scans denoised by neural networks look similar to small capillaries and might therefore represent information which is lost in multi-frame averaging. However, confirmation that these represent actual structures would require complementary OCT angiography, which is currently not available for the dataset. The quantitative performance of our approach exceeded that of the non-local filtering baselines BM3D^13^ and PNLM^14^. Compared to Gaussian filtering, neural network based denoising leads to considerably less blurring and better contrast in a similar time frame, although, in terms of PSNR and SSIM, the neural network did not outperform Gaussian filtering. While showing limitations to achievable quality under the given circumstances, these numerical results must be treated carefully. Using averaged, registered frames as ground truth inherently rewards some degree of blurring, especially since it commonly relies on frames from slightly different positions to reduce structure-dependent speckle.

Self-supervised training of blind-spot networks has very little demands on the training data—a set of unpaired noisy images is sufficient. However, the blind-spot characteristic itself inherently limits the achievable image quality and resolution by completely ignoring potentially useful information for predicting individual pixel values due to the masking—including the corrupted pixel value itself. On the other hand, sufficient masking is crucial to fully remove all noise. This may have been the cause for the residual noise particularly appearing within tissue when denoised with the most complex networks. Due to the relatively dense lateral sampling of training images, residual information about speckle might be present in adjacent A-scans, allowing partial recovery of these patterns given sufficient network complexity. The better results for smaller networks indicate that, by decreasing the number of channels in all layers including the lowest, the U-Net becomes more robust to such masking imperfections. This is underlined by the performance increase when omitting the uppermost skip-connection, which prevents the information flow from bypassing the deeper layers. This performance gain comes however at a cost of an increased blurring and loss of detail. Given these challenges, future work might aim to further improve the denoising quality given the small network size by further optimizing the network architecture as well as training strategy. In principle, depending on available data, any learning method ranging from unsupervised to various forms of fully supervised training could be used. Especially the latter might further improve the image quality for an identical network architecture.

An interesting perspective may involve artifact reduction as an additional training goal. Currently, artifacts are not explicitly removed as long as their appearance and spatial extent differs from the noise. This can increase the visibility and therefore impact of, for example, detector saturation artifacts when lowering the rendering noise floor to fully appreciate the effect of denoising. We are planning to combine denoising with artifact reduction in future work to address this effect.

Although the focus of this work clearly was the real-time implementation and application of denoising, we could show that, using selected optimizations proposed in N2V2^28^, high-frequency artifacts can be reduced for structured Noise2Void^27^ as well, leading to increased numerical denoising performance. At the same time, these changes also benefit the real-time applicability by reducing inference speed. Especially removing the top-level skip connection from the U-Net benefits the achievable throughput, since it is associated with costly concatenation as well as additional convolution operations within the network. The residual connection on the other hand, requiring only a single element-wise addition, did not have a large impact on the measured inference speed.

Integration of a fully trained and easily replaceable network into the reconstruction pipeline allows for large flexibility, not only with respect to architecture and training method. Given this flexibility, we believe that this work could set the foundation for various further real-time AI applications in 4D-OCT relying on full-sized data, including, but not limited to, further image quality enhancements. For example, although we observed our models to generalize well across scenarios and even OCT devices, it may be beneficial to train networks specifically for certain use-cases: Separately optimized models for visualization of the retina or cornea could potentially better account for their unique characteristics, including differences in SNR due to distinct optical properties of the individual segments of the eye. With paired training data, future work may build upon this flexibility and compare other training approaches, including but not limited to Noise2Noise^21^ and supervised approaches, to train similar networks, which could then easily be incorporated to potentially further improve the visualization quality.

Technological advancements in soft- and hardware, specifically GPUs, will allow faster inference times, further increasing the possibilities for real-time volumetric denoising. However, it can also be expected that OCT-systems will become faster as laser and data acquisition technology advances, exacerbating the timing constraints. Depending on such developments, future work may investigate the feasibility of using 3D convolutional networks which could exploit all available spatial information. For the time being, direct application of 3D networks is unfeasible not only because of processing speed—limiting throughput—but also because it would inherently introduce unacceptable latency. This is due to the requirement to wait for an entire volume acquisition to finish before being able to process it. A potential approach to reduce this limitation could be to process partial volumes, but dividing the volume might be difficult given the currently used spiral scanning. Future work may aim to include spatio-temporal information from the volume series to improve denoising robustness.

## Supplementary Information

Supplementary Information 1.

Supplementary Video 1.

Supplementary Video 2.

Supplementary Video 3.

Supplementary Video 4.

## Data Availability

The datasets generated and/or analyzed during the current study are not published, but may be made available from the corresponding author upon reasonable request.

## Code availability

The code to generate the results of this study is not publicly available, but training code can be made available upon reasonable request.
