# Supplementary material for: Live 4D-OCT denoising with self-supervised deep learning
Source: Sci Rep. 2023 Apr 8;13:5760. doi: 10.1038/s41598-023-32695-1 (PMC10082772; doi:10.1038/s41598-023-32695-1)
Supplement: Supplementary file 1 — Supplementary Information 1. [file 41598_2023_32695_MOESM1_ESM.pdf]

Supplementary material for

Live 4D-OCT denoising with self-supervised deep learning

Jonas Nienhaus[1,*], Philipp Matten[1], Anja Britten[1], Julius Scherer[1], Eva Höck[2], Alexander Freytag[2], Wolfgang Drexler[1], Rainer A. Leitgeb[1], Thomas Schlegl[1,+], and Tilman Schmoll[1,3,+]

[1]Medical University of Vienna, Center for Medical Physics and Biomedical Engineering, Austria
[2]Carl Zeiss AG, Germany
[3]Carl Zeiss Meditec, Inc., USA
[*]jonas.nienhaus@meduniwien.ac.at
[+]these authors jointly supervised this work

This file contains supplementary material not included in the paper itself. In addition to this document, four videos are provided as supplementary material. A general description and individual video captions for these files are given in section 1 of this document. In section 2, the full evaluation dataset, highlighting important regions of interest, is presented. Example B-scans from the training dataset are provided in section 3. Section 4 contains additional comparisons of denoised evaluation images, similar to the example shown in the main publication.

1 Video captions

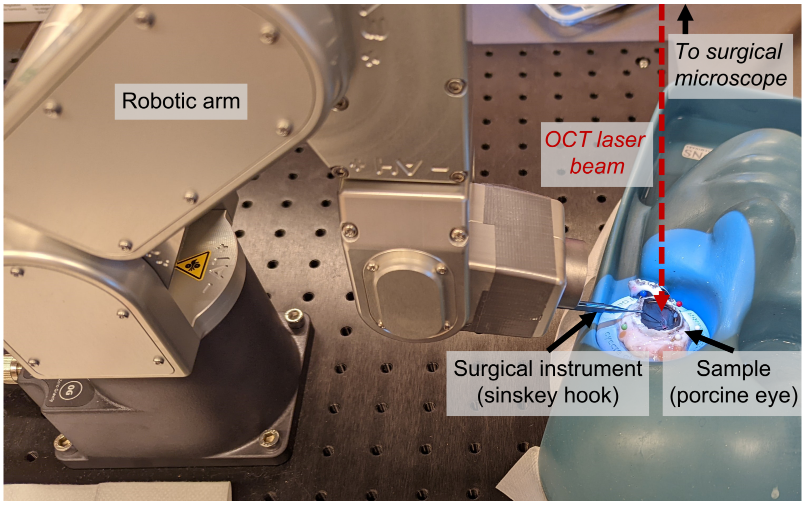

Figure S1. Picture of the setup used during acquisition. A Sinskey hook surgical instrument is attached to a robotic arm (Meca500, Mecademic Robotics, Montreal, Canada) and placed beneath a surgical microscope (ARTEVO 800, ZEISS, Jena, Germany; not shown) coupled to the 4D-OCT prototype. For retinal imaging, the porcine eyes were cut open, as shown in the picture.

The additional supplement includes three videos showing OCT-visualizations with and without denoising applied during live imaging. All videos show a side-by-side view of comparable sequences performed on ex-vivo porcine eyes. All compared sequences were acquired separately, but within few minutes in time. For the 4D modes, noise floor level for the rendering in CAMPVis[1,2] are displayed (in the range $[0,1]$). Mock surgical maneuvers with different movement speeds were performed by a robotic arm (Meca500, Mecademic Robotics, Montreal, Canada) or manually by the authors. They were not aimed to represent realistic interventions, but to allow visualization of representative dynamic and reproducible instrument-tissue interactions. The basic setup with the Sinskey hook instrument attached to the robot and an opened porcine eye is shown in Figure S1. The following four videos are attached:

- **Supplementary Video 1**: Comparison of instrument maneuvers in the 2D mode performed manually on the cornea. The original display showed B-scans with 40 frames per second (fps), the video file shows a screen capture with 60 fps resampled to 40 fps. The B-scans are displayed with identical scale settings, i.e. subtracted blacklevel and scaling factor.
- **Supplementary Video 2**: Comparison of the posterior 4D mode, rendered with 10 volumes per second (vps) and screen-captured with 20 fps. The file contains five sequences showing maneuvers exclusively performed by the robot. The first video sequence corresponds to the volume series presented in the paper. For imaging of the retina, the porcine eye was cut open.
- **Supplementary Video 3**: Comparison of the anterior 4D mode, rendered with 3 vps and screen-captured with 20 fps. The file contains three sequences showing maneuvers exclusively performed by the robot. The first video sequence corresponds to the volume series presented in the paper.
- **Supplementary Video 4**: Renderings of the anterior segment, but using the posterior mode with 10 vps and a reduced field of view compared to the anterior mode. The file contains seven sequences showing maneuvers exclusively performed by the robot, including sequences where the instrument penetrates the anterior chamber.

2 Evaluation dataset

The entire evaluation dataset is shown in Figures S2, S3 and S4. All images were acquired using a PLEX Elite 9000 (ZEISS, Dublin, CA, USA). Averages of 100 registered frames were used as ground truth. SSIM and PSNR were evaluated for regions marked in yellow. The CNR was evaluated for single background regions (blue) and 14 foreground regions (red) in every image, roughly covering seven different individual layers in the retina. All images, as well as all regions of interest for the CNR, were weighted equally to determine the final scores.

3 Training and validation datasets

Examples from the training set are shown in Figure S5. Based on the noise autocorrelations, we selected to mask an area of 7×1 pixels. Figure S6 shows examples from the validation set.

4 Denoised evaluation images

Additional denoised evaluation images, upon which the reported numerical scores are based, are shown in Figures S7-S9. In all images, U-Net^{r,t} denotes a standard U-Net[3] with depth 2 and a residual connection. In U-Net$^{\neg r,t}$ no residual connection was used, while for U-Net$^{\neg r,\neg t}$, also the top-level skip connection was omitted.

References

1. Schulte zu Berge, C., Grunau, A., Mahmud, H. & Navab, N. Campvis - a game engine-inspired research framework for medical imaging and visualization. Tech. Rep., Technische Universität München (2014).
2. Weiss, J. *et al.* Layer-Aware iOCT Volume Rendering for Retinal Surgery. In Kozlíková, B., Linsen, L., Vázquez, P.-P., Lawonn, K. & Raidou, R. G. (eds.) *Eurographics Workshop on Visual Computing for Biology and Medicine*, DOI: 10.2312vcbm.20191239 (The Eurographics Association, 2019).
3. Ronneberger, O., Fischer, P. & Brox, T. U-net convolutional networks for biomedical image segmentation. In *International Conference on Medical image computing and computer-assisted intervention*, 234–241 (Springer, 2015).

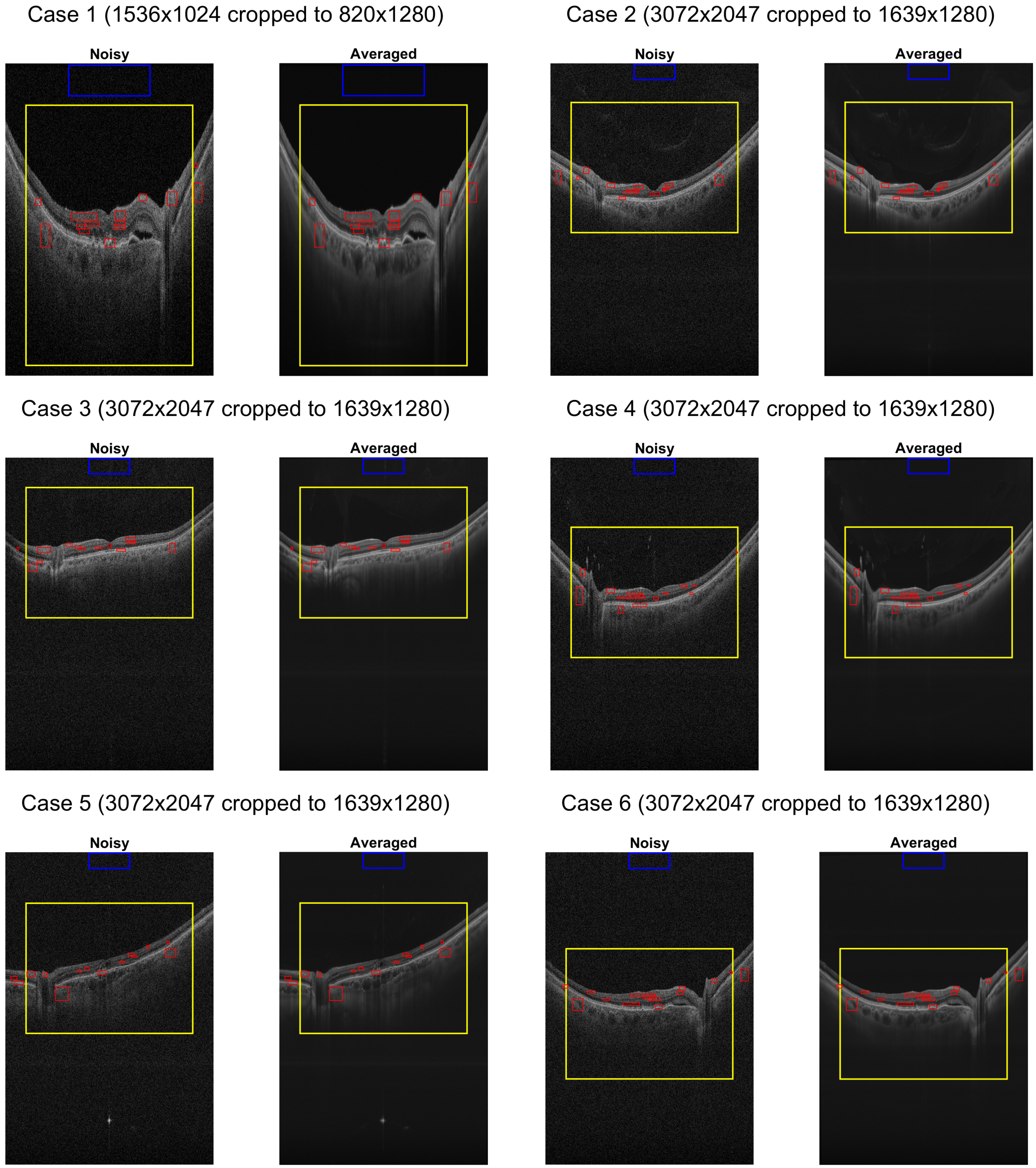

Figure S2. First 6 B-scans used for numerical evaluation.

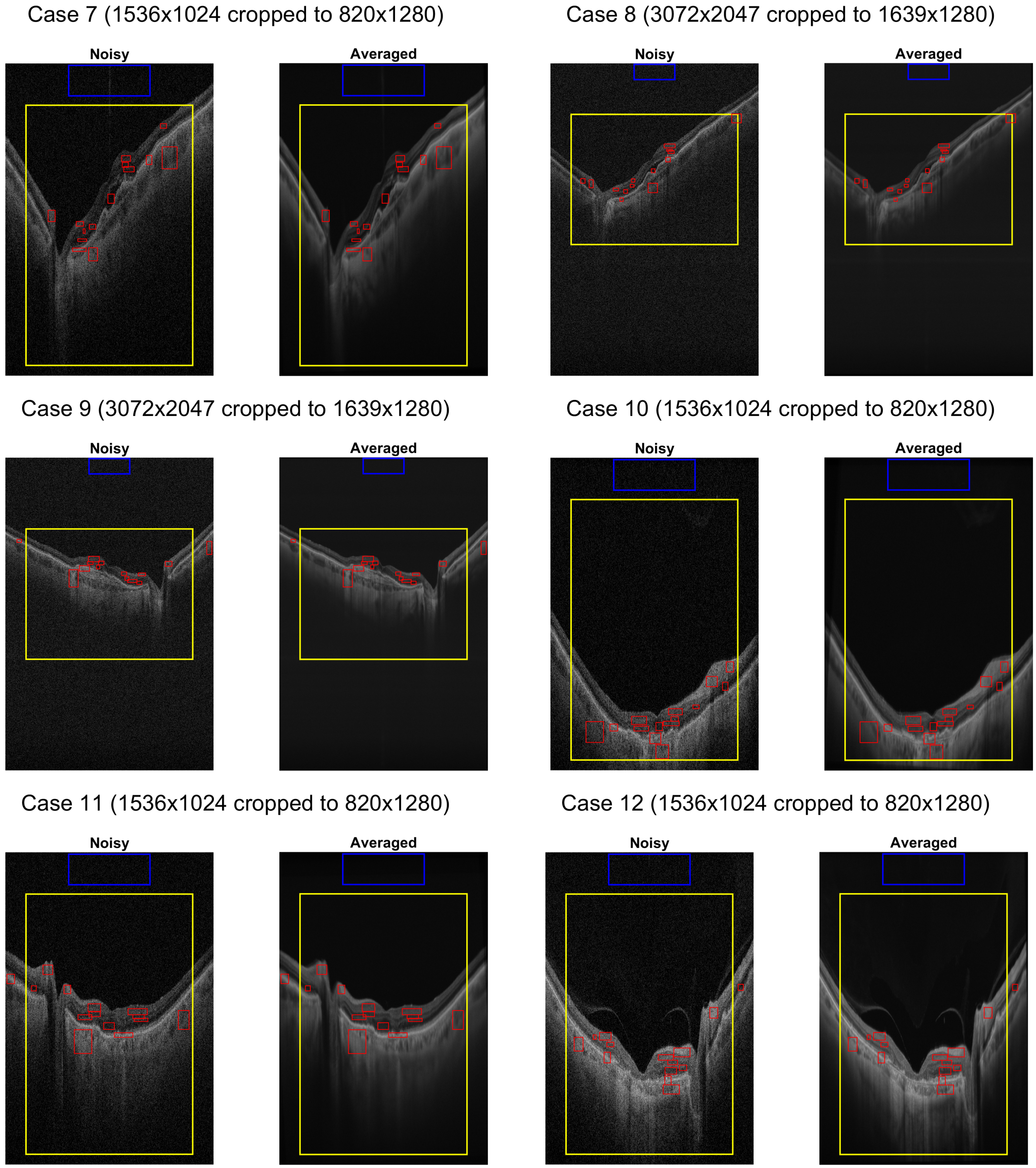

Figure S3. B-scans 7-12 used for numerical evaluation.

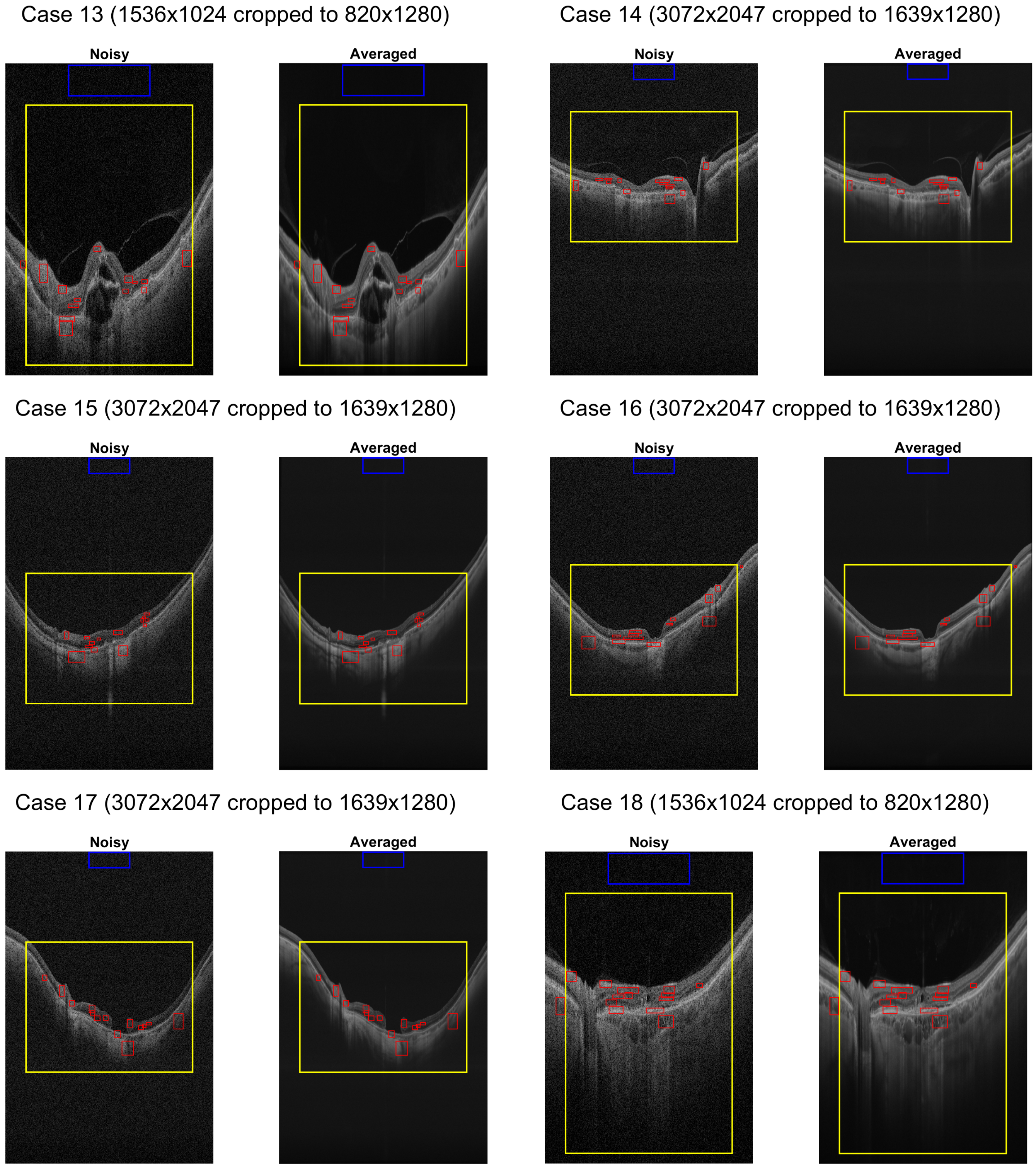

Figure S4. B-scans 13-18 used for numerical evaluation.

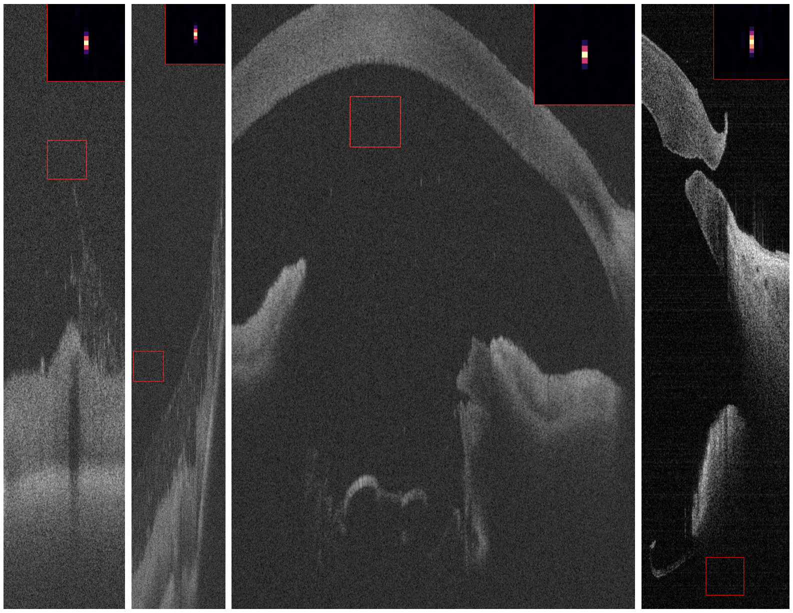

(a) B-scans of bovine retinas and porcine anterior segments (both ex-vivo), acquired with the 4D-OCT prototype.

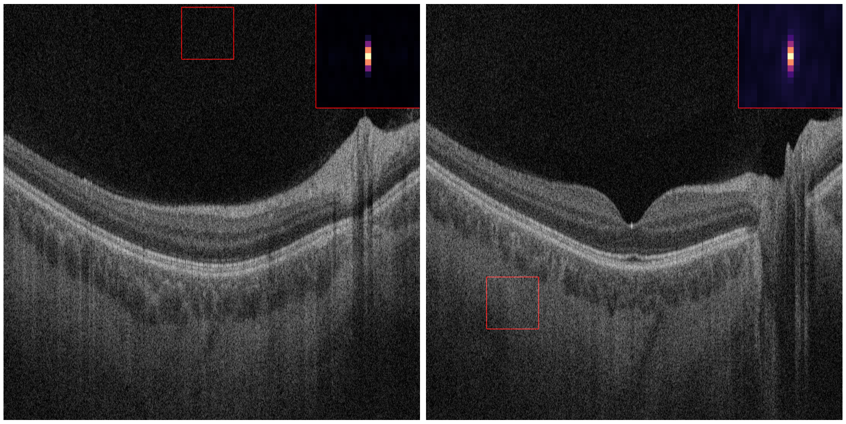

(b) B-scans of a human retina, acquired with a PLEX Elite.

Figure S5. Examples from the training set. The spatial autocorrelation for the marked (red boxes) selected homogeneous regions of interest is displayed in the top right corners.

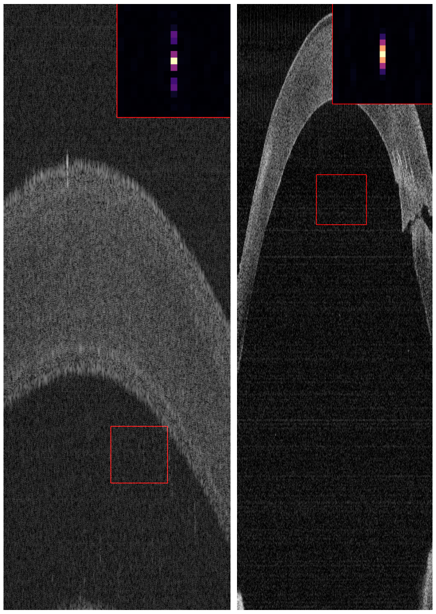

(a) B-scans of bovine and porcine anterior segments (both ex-vivo), acquired with the 4D-OCT prototype.

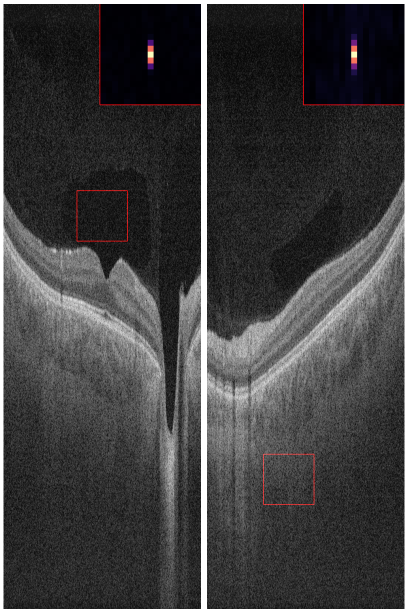

(b) B-scans of a human retina, acquired with a PLEX Elite.

Figure S6. Examples from the validation set. The spatial autocorrelation for the marked (red boxes) selected homogeneous regions of interest is displayed in the top right corners. Only the spatial noise autocorrelation within the training set was used to determine the mask size.

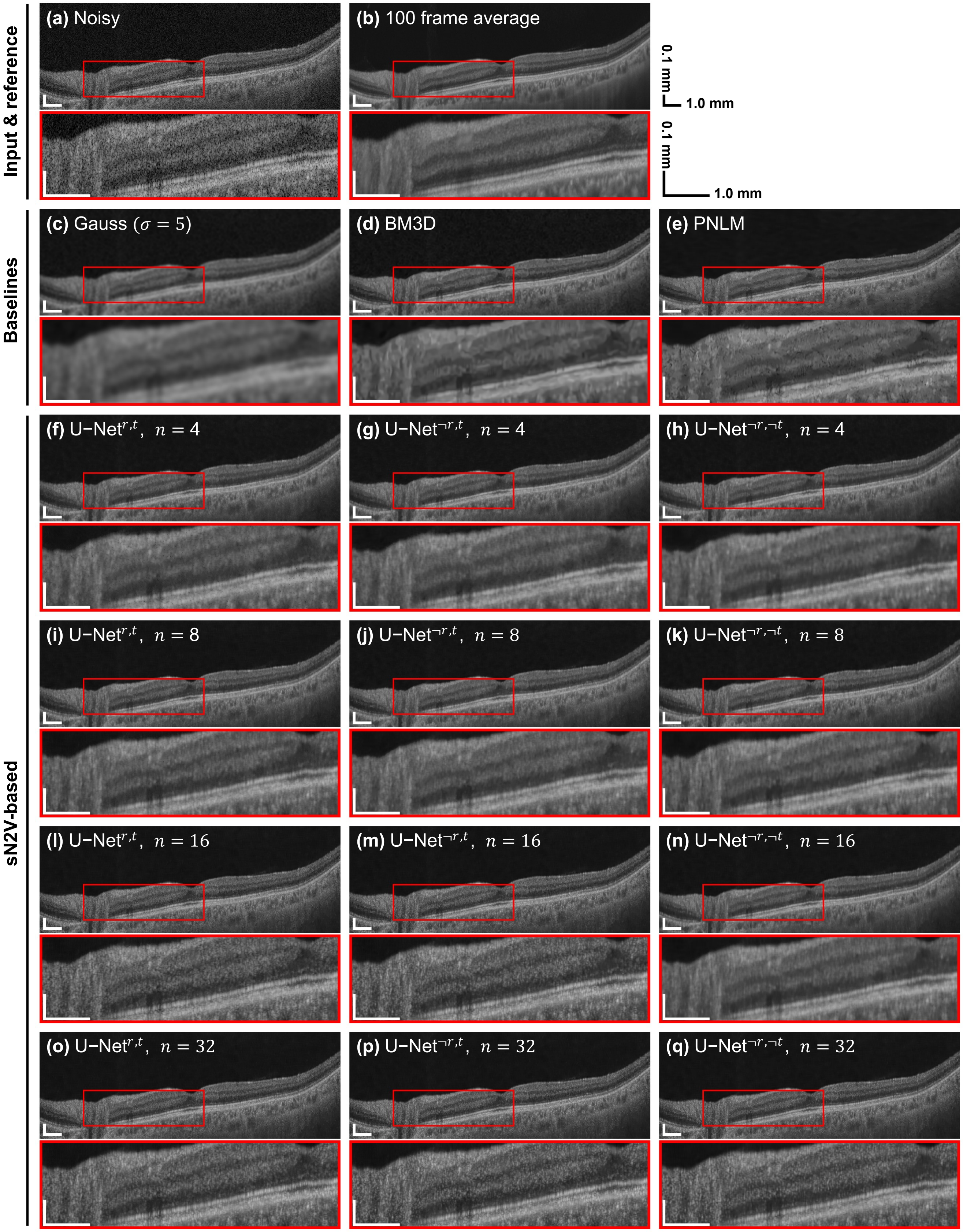

Figure S7. Original, averaged, filtered and denoised frames of case 3.

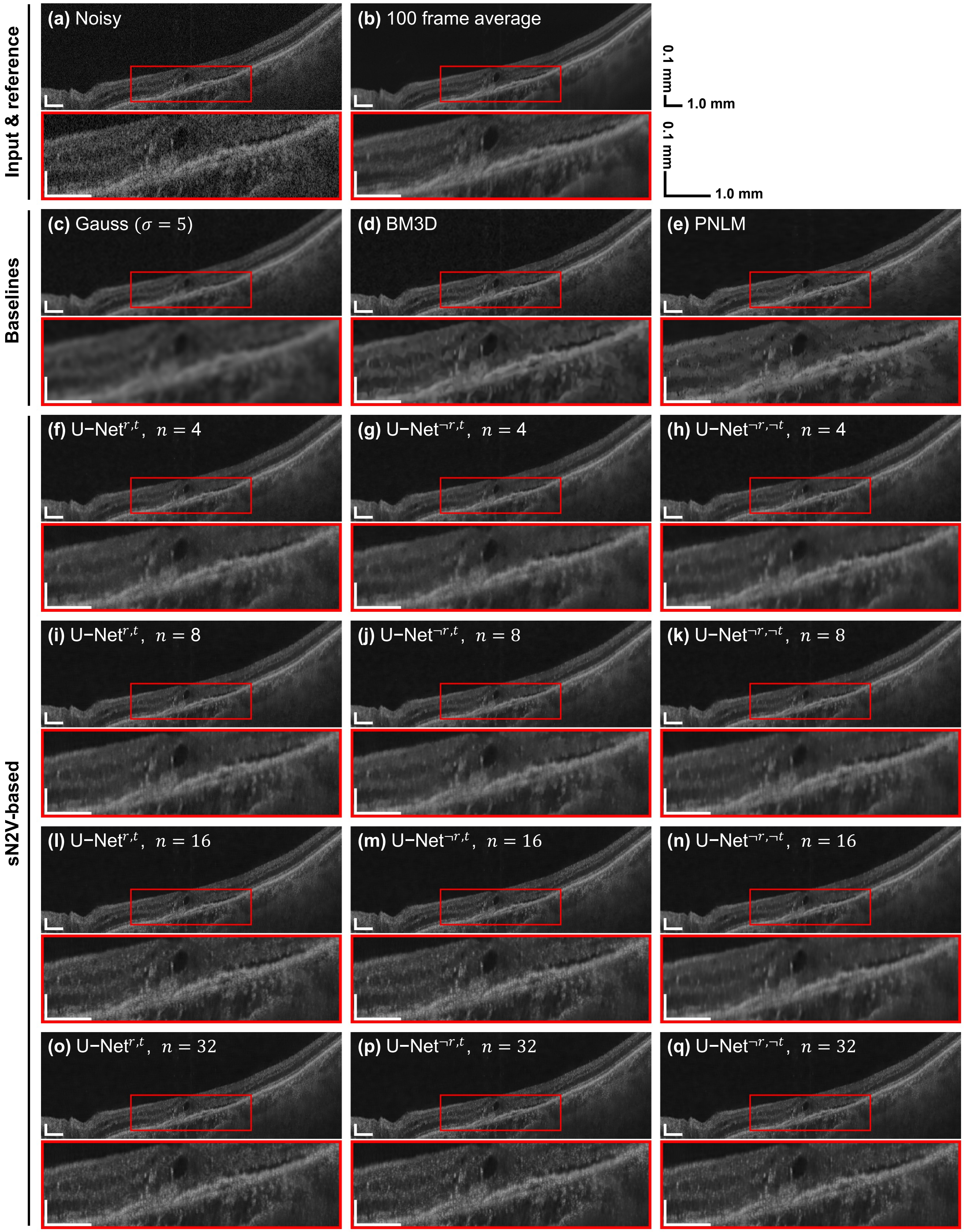

Figure S8. Original, averaged, filtered and denoised frames of case 5.

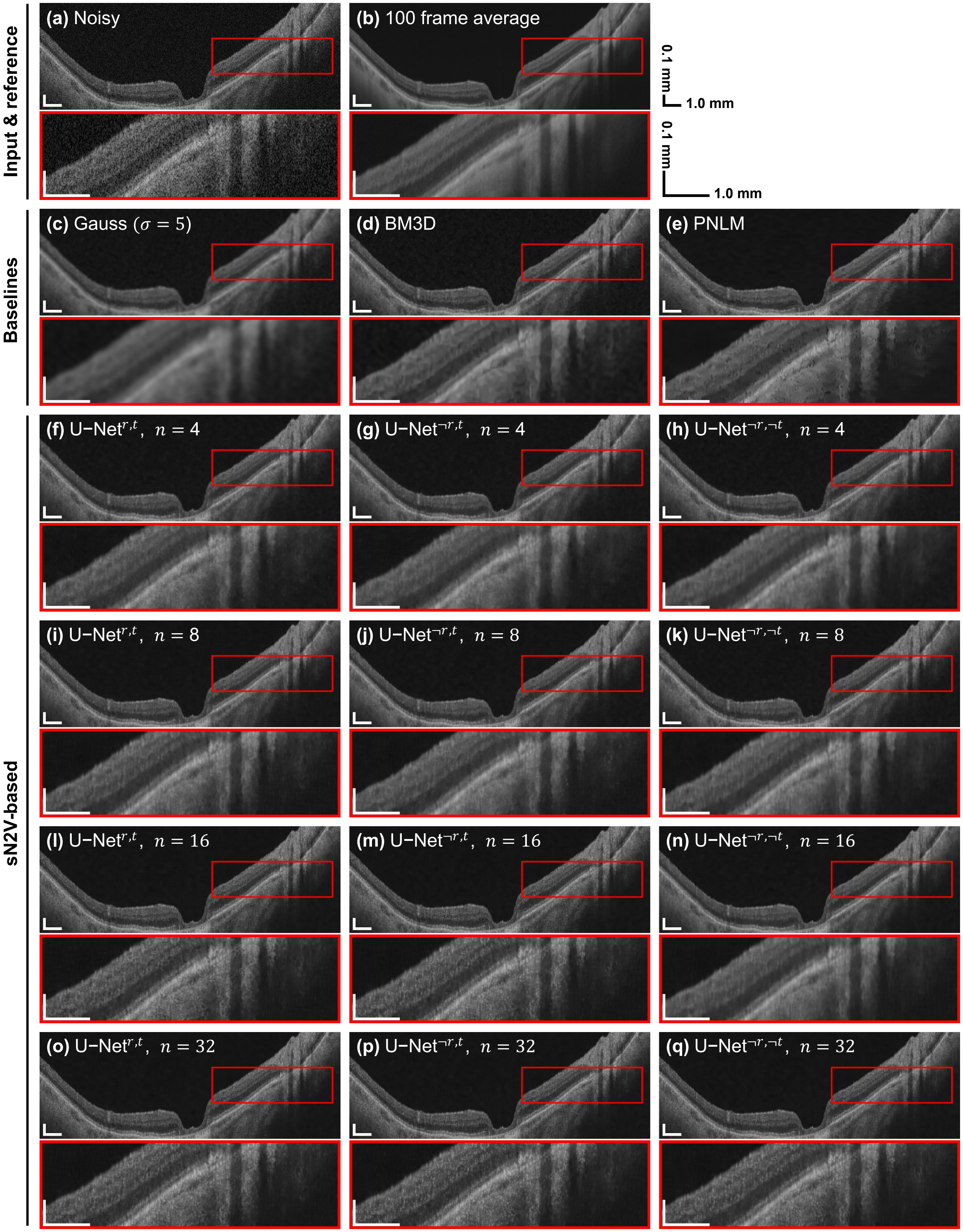

Figure S9. Original, averaged, filtered and denoised frames of case 16.
